# Two hepatic cavernous hemangiomas mimicking colorectal liver metastasis: A rare case report

**DOI:** 10.1016/j.ijscr.2021.105817

**Published:** 2021-03-23

**Authors:** Tlal Matouq Alsofyani, Mohammed Yousef Aldossary, Faisal Fahd AlQahtani, Mahmoud Tabbal, Ameera Balhareth

**Affiliations:** a Department of General Surgery, Colorectal Surgery Section, King Fahad Specialist Hospital-Dammam, Dammam, Saudi Arabia; b Department of General Surgery, King Abdulaziz Hospital, National Guard Hospital, AlAhsa, Saudi Arabia; c Department of General Surgery, Hepatobiliary Surgery Section, King Fahad Specialist Hospital-Dammam, Dammam, Saudi Arabia

**Keywords:** Sigmoid cancer, Liver metastasis, Cavernous hemangioma

## Abstract

**Introduction:**

1 Hepatic cavernous hemangioma (HCH) is a common benign lesion of the liver. The radiological features of HCH can resemble those of hepatic malignancies such as metastatic liver cancer, which make the preoperative definitive diagnosis difficult.

**Presentation of case:**

We report the case of a 77-year-old woman who presented with a 2-month history of abdominal pain and per rectum fresh bleeding. The initial diagnosis at the referral hospital, based on computed tomography (CT) scan of the chest, abdomen, and pelvis, was sigmoid colon cancer with liver metastasis. Further evaluation at our hospital, the patient underwent colonoscopy and biopsy. Histopathological examination of the biopsy revealed an invasive moderately differentiated adenocarcinoma. The patient underwent laparoscopic sigmoidectomy and left hepatectomy. The histopathological examination of the sigmoid colon mass revealed an invasive moderately to poorly differentiated adenocarcinoma. One out of twenty lymph nodes is positive for metastatic carcinoma. The pathological stage is pT3, pN1a, pM0. The histopathological examination of the left hepatic lobe revealed two hyalinized cavernous hemangiomas with no malignancy is seen. The postoperative recovery was uneventful, and the patient was discharged home with regular follow-up in our outpatient clinic.

**Discussion:**

HCH is congenital vascular malformation and is the most common benign hepatic tumors. Fine-needle biopsy during diagnostic laparoscopy for undiagnosed multiple liver tumor can differentiate atypical HCH from colorectal metastasis but can potentially lead to rupture or seeding of cancer cells.

**Conclusion:**

Surgical resection is necessary to determine if the mass is malignant in some atypical HCH mimicking liver metastasis to confirm the diagnosis with histopathologic examination.

## Highlights

• Hepatic cavernous hemangioma is a common benign lesion of the liver.
• Commonly seen in women and most of the patients are asymptomatic.
• The radiological features can resemble those of hepatic malignancies, which make the preoperative definitive diagnosis difficult.
• Surgical resection is necessary to confirm the diagnosis.

## 1 Introduction

HCH is a common benign lesion of the liver and usually occurs more in females [1]. The size of these lesions is usually larger than 3 cm and most of the patients are asymptomatic [2]. Large lesions of HCH may present with a variety of symptoms, including abdominal pain, mass, jaundice, hemorrhage, nausea, and vomiting [3,4]. The radiological features of HCH can resemble those of hepatic malignancies such as metastatic liver cancer, fibrolamellar hepatocellular carcinoma, intrahepatic cholangiocarcinoma, which make the preoperative definitive diagnosis difficult. Here, we report a case of HCH mimicking colorectal liver metastasis. This case report has been reported in line with the SCARE criteria [5].

## 2 Presentation of case

A 77-year-old woman, known to have diabetes mellitus and hypertension on treatment. The patient was referred to our regional hospital as a case of sigmoid cancer with liver metastasis. The patient was complaining of generalized moderate abdominal pain for 2 months. The pain was intermittent, not increased with meals, and was not radiating. She gave a history of intermittent fresh per rectum bleeding for 2 months. Also, she had a long history of constipation for 7 years on intermittent laxative medications. She denied any history of fatigue, anorexia, diarrhea, jaundice, and weight loss. Also, the patient denied any history of smoking or alcohol intake. The past surgical history and family history were unremarkable.

**Fig. 1:**
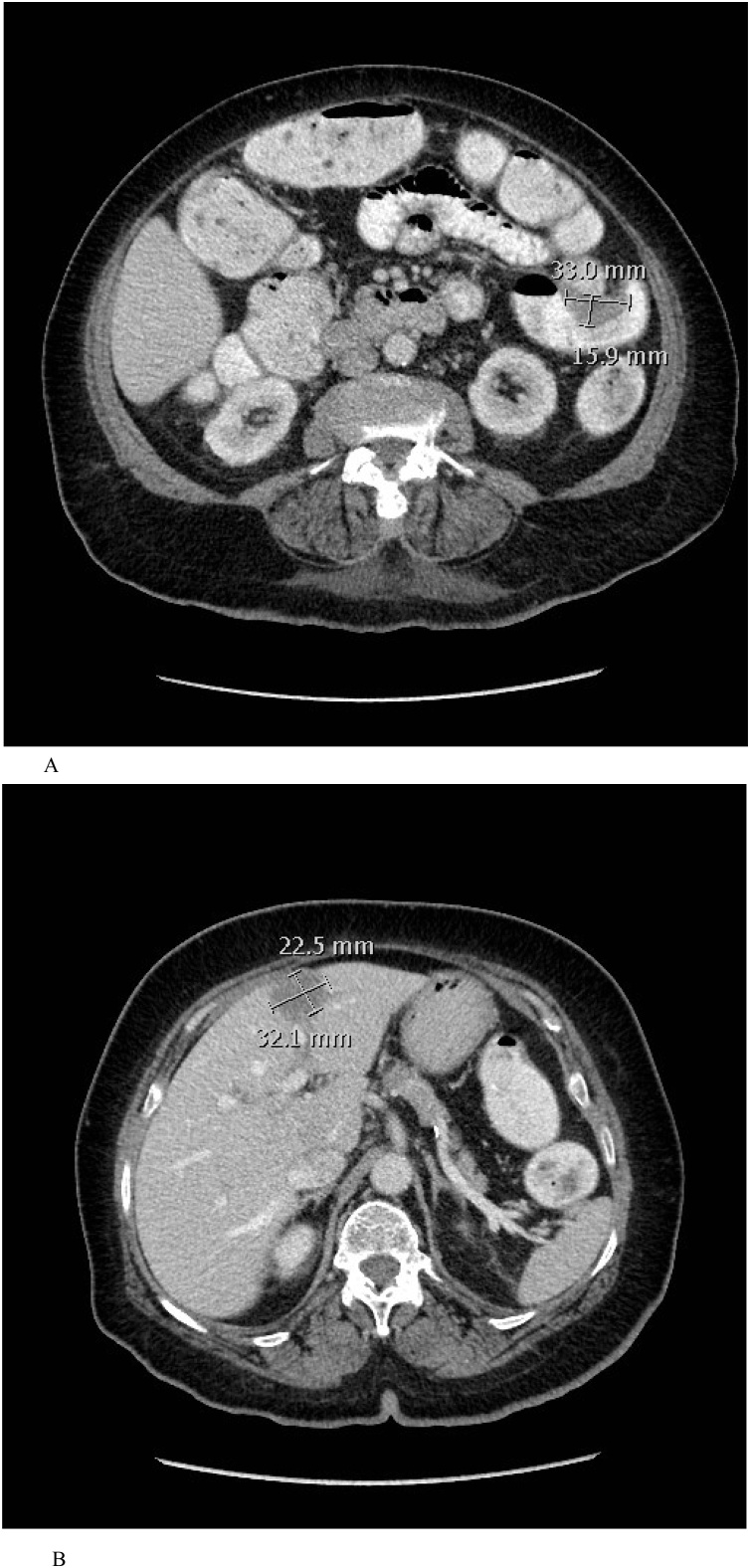
A: CT scan of the chest, abdomen, and pelvis with contrast showed a focal polypoidal thickening seen at the proximal sigmoid colon extending 3 cm long with a maximum wall thickness of 2 cm. B: CT scan of the chest, abdomen, and pelvis with contrast showed a few hypodense nodules in the liver, the largest in segment IV, measuring 3.4 × 2.7 cm, and highly suspicious for liver metastasis.

**Fig. 2:**
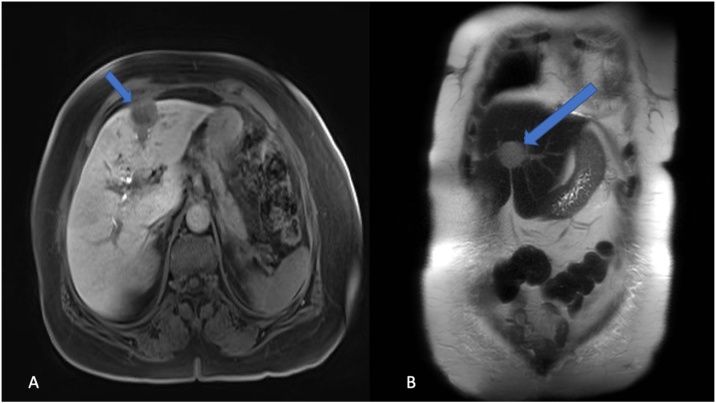
A and B: MRI of abdomen showed two left hepatic lesions located between the medial and lateral portions of the left hepatic lobe (segment IV and II/III), the largest measuring 2.7 × 2.4 × 2.8 cm, and highly suspicious of colorectal liver metastasis.

Upon general physical examination, the patient not in pain, and had no pallor or jaundice. She was vitally stable and afebrile. Her abdomen was soft, not tender or distended, and no palpable masses were felt. Per rectum examination with anoscopy revealed an empty rectum with no fissure or hemorrhoid can be seen. Laboratory investigations showed neither leukocytosis nor neutrophilia. Other laboratory results were unremarkable. The cancer antigen markers including carcinoembryonic antigen, carbohydrate antigen 19.9, and α-fetoprotein were all within normal ranges. CT scan of the chest, abdomen, and pelvis with contrast performed in the referring hospital showed a focal polypoidal thickening seen at the proximal sigmoid colon extending 3 cm long with a maximum wall thickness of 2 cm (Fig. 1A). There are a few hypodense nodules in the liver, the largest in segment IV, measuring 3.4 × 2.7 cm, and highly suspicious for liver metastasis (Fig. 1B). Further evaluation of liver nodules, magnetic resonance imaging (MRI) of abdomen was performed and revealed two left hepatic lesions located between the medial and lateral portions of the left hepatic lobe (segment IV and II/III), the largest measuring 2.7 × 2.4 × 2.8 cm showing intermediate high T2 and low T1 signal intensity, with faint peripheral rim enhancement in all phases with no central filling in delayed phase, and highly suspicious of colorectal liver metastasis (Fig. 2A and B). The case was discussed in multidisciplinary team and the decision was to do a colonoscopy to examine the colon, and to take a biopsy. The colonoscopy findings showed a polypoidal lesion seen in the proximal sigmoid colon, not obstructing, no other lesions can be seen at the rest of the colon, and biopsy was taken. Histopathological examination of the sigmoid colon biopsy revealed an invasive moderately differentiated adenocarcinoma. The hepatobiliary surgery team was consulted for evaluation the patient and the possibility to respect the liver metastases.

**Fig. 3:**
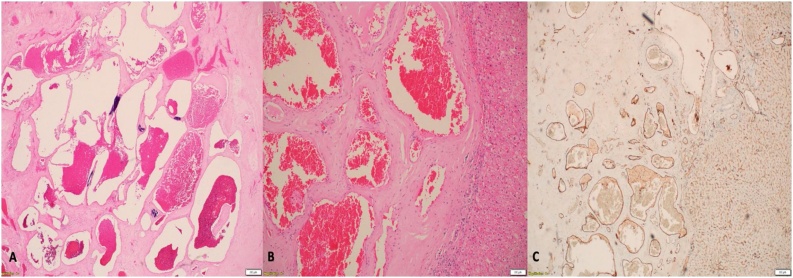
A, B, and C: **A:** Haemotoxylin and eosin staining showing variably sized vascular spaces lined by flattened endothelial cells. **B**: These spaces are separated by hyalinized fibrous stroma with calcification (magnification 4×). **C**: Immunoreactivity of the lining endothelial cells for CD31 (magnification 4×).

The decision was made to take the patient to the operation room. Laparoscopic sigmoidectomy and colorectal anastomosis was performed. The hepatobiliary surgery team joined us in operation room after the sigmoid resection. An Intraoperative ultrasound of the liver was performed and showed two lesions in the left lobe involving segment IVB and III. The decision was made to perform a left hepatectomy and liver frozen section revealed negative margin. The gross examination of the sigmoid mass revealed a tumor measuring 3 × 3 cm and the histopathological examination of the sigmoid colon mass revealed an invasive moderately to poorly differentiated adenocarcinoma. The tumor invades through the muscularis propria into pericolorectal tissue. No lymphovascular or perineural invasion identified. One out of twenty lymph nodes are positive for metastatic carcinoma and all margins uninvolved by tumor. The pathological stage is pT3, pN1a, pM0. The microscopic examination of the left hepatic lobe revealed two hyalinized cavernous hemangiomas with no malignancy is seen (Fig. 3A–C). The postoperative recovery was uneventful. The patient was discharged home in a good condition with regular follow-up during the last 8 months post operation in our outpatient clinic. The patient was referred to medical oncology and received six cycles of adjuvant capecitabine (Xeloda) without any complications. CT scan of the chest, abdomen, and pelvis was performed during his follow-up and showed no evidence of local recurrence or distant metastasis.

## 3 Discussion

HCH is congenital vascular malformation and is the most common benign hepatic tumors [1,6]. Most of the patients are usually asymptomatic [2]. Abdominal pain or discomfort, jaundice, hemorrhage, and nausea, were the most common presenting symptoms [[3], [4], [5], [6]]. Invasive management of symptomatic hepatic hemangioma mainly consists of surgery and interventional radiology, including trans-arterial embolization, ablation and percutaneous sclerotherapy [7]. In our study, HCH was misdiagnosed as colorectal liver metastasis. All the radiological findings were compatible with liver metastasis as found in the literature. Moon HK et al. [1] reported hepatic hemangioma misdiagnosed as gastric submucosal tumor demonstrating the atypical CT findings of hepatic hemangioma. Yamada et al. [8] reported a hepatic sclerosed hemangioma which was misdiagnosed as metastasis of gastric cancer. Another three cases reported hepatic sclerosing hemangioma mimicking metastatic liver tumor from colorectal cancer [[9], [10], [11]]. The radiological diagnosis of typical HCH is usually established by CT scan and MRI. However certain atypical cases may make the diagnosis difficult [12]. Multiple characteristics of the atypical hemangiomas described in the literature including giant, heterogeneous uptake, rapidly filling, calcified, hyalinized, cystic, or pedunculated, and these cases will require histopathologic examination [13,14]. Fine-needle biopsy during diagnostic laparoscopy for undiagnosed multiple liver tumors can differentiate atypical HCH from colorectal metastasis, but can potentially lead to rupture or seeding of cancer cells [10,15]. Atypical HCH can be treated by surgery (hepatic resection or enucleation, open, laparoscopic, or robotic) according to number location, relation to the blood supply, and surgical experience [16]. Surgery is necessary to determine if the mass is malignant in some atypical HCH mimicking liver metastasis to confirm the diagnosis with histopathologic examination.

## 4 Conclusion

HCH mimicking liver metastasis despite accurate preoperative investigation. HCH needs a high index of suspicion among the differential diagnoses of multiple liver tumors in patients with colorectal cancer.

## Declaration of Competing Interest

The authors declare no conflict of interest.

## Sources of funding

This study did not receive any funding from governmental or private organizations.

## Ethical approval

Ethical approval was obtained from the Institutional Review Board of the King Fahad Specialist Hospital, Dammam, Saudi Arabia. The reference number (IRB- Pub-04-021) Dated 08 March 2021.

## Consent

Written informed consent was obtained from the patient for publication of this case report and accompanying images. A copy of the written consent is available for review by the Editor-in-Chief of this journal on request.

## Author contribution

Study concept or design – AB, TMS, MT.

Data collection – AB, TMS, MYD, FFQ.

Data interpretation – TMS, MYD, FFQ.

Literature review – TMS, MYD, FFQ.

Drafting of the paper – TMS, MYD, FFQ, AB.

Editing of the paper – TMS, MYD, AB.

## Registration of research studies

Not required.

## Guarantor

Ameera Balhareth.

## Provenance and peer review

Not commissioned, externally peer-reviewed.
